# Effects of aerobic and resistance exercise on glycosylated hemoglobin (HbA1c) concentrations in non-diabetic Taiwanese individuals based on the waist-hip ratio

**DOI:** 10.1371/journal.pone.0267387

**Published:** 2022-05-05

**Authors:** Ying-Hsiang Chou, Yung-Yin Cheng, Oswald Ndi Nfor, Pei-Hsin Chen, Che‐Hong Chen, Hsin-Lin Chen, Bo-Jiun Chang, Disline Manli Tantoh, Chien-Ning Huang, Yung-Po Liaw

**Affiliations:** 1 Department of Radiation Oncology, Chung Shan Medical University Hospital, Taichung, Taiwan; 2 School of Medicine, Chung Shan Medical University, Taichung, Taiwan; 3 School of Medical Imaging and Radiological Sciences, Chung Shan Medical University, Taichung, Taiwan; 4 Department of Medical Imaging, Chung Shan Medical University Hospital, Taichung, Taiwan; 5 Department of Public Health and Institute of Public Health, Chung Shan Medical University, Taichung, Taiwan; 6 Department of Chemical and Systems Biology, Stanford University School of Medicine, Stanford, CA, United States of America; 7 Department of Internal Medicine, Chung Shan Medical University Hospital, Taichung, Taiwan; 8 Institute of Medicine, Chung Shan Medical University, Taichung, Taiwan; The University of Mississippi Medical Center, UNITED STATES

## Abstract

**Background:**

Glycosylated hemoglobin (HbA1c) reflects the average blood sugar over the past eight to twelve weeks. Several demographic and lifestyle factors are known to affect HbA1c levels. We evaluated the association of HbA1c with aerobic and resistance exercise in non-diabetic Taiwanese adults based on the waist-hip ratio (WHR).

**Methods:**

We conducted this study based on TWB data collected from 90,958 individuals between 2008 and 2019. We estimated the Beta (β) coefficient and 95% confidence intervals (CI) for HbA1c using multivariate regression models.

**Results:**

Based on the multivariate analysis, lower HbA1c levels were associated with both resistance exercise (β-coefficient = -0.027, 95% CI -0.037 to -0.017) and aerobic exercise (β-coefficient = 0.018, 95% CI, -0.023 to -0.013). Higher HbA1c levels were associated with abnormal WHR compared to normal WHR (β-coefficient = 0.091, 95% CI, 0.086 to 0.096). We detected an interaction between exercise and WHR (p for interaction = 0.0181). To determine the magnitude of the interaction, we performed additional analyses (with the reference group being ’abnormal WHR with no exercise’) and observed substantial decreases in HbA1c regardless of the WHR and exercise category. However, the largest reduction occurred in the ’normal WHR and resistance exercise’ group (β = -0.121, 95% CI, -0.132 to -0.109).

**Conclusions:**

We found that normal resistance exercise, coupled with a normal WHR was significantly associated with lower HbA1c levels among non-diabetic individuals in Taiwan.

## Introduction

Hemoglobin A1c, formed by the nonenzymatic glycosylation of hemoglobin is essential for monitoring the management of chronic diabetes [1]. It is the main fraction among the various glycated hemoglobins and appears to be affected by iron deficiency and vitamin B12 deficiency [2]. Its measurement is essential for long-term glucose control, where a concentration of less than 7% is considered the target for good control in most cases with diabetes [3]. According to the 2009 International Expert Committee Report on the Role of the A1C Assay in the Diagnosis of Diabetes, HbA1c is considered a more stable biological index than fasting plasma glucose (FPG) and oral glucose tolerance test (OGTT) [4, 5].

Associations have been reported between HbA1c levels and cardiovascular diseases in both diabetic and non-diabetic patients [6–9]. Individuals with elevated HbA1c concentrations are more likely to develop complications related to diabetes such as coronary lesions [10]. Harvey and colleagues observed a significant decrease in major adverse cardiovascular events (MACE) among individuals with diabetes who had HbA1c levels below 7% [11]. Mortality associated with diabetes mellitus was reportedly 15.3% higher among patients who had HbA1c levels above 8% than in those with levels below 6% [12].

Besides glucose, demographic factors including age, sex, body mass index, and other variables have been investigated for HbA1c variability [13]. Results pointed to BMI and sex-specific differences in HbA1c variability among the study participants. In epidemiological studies, WHR and BMI were positively related to HbA1c among cases with prediabetes [14] and type 2 diabetes [15]. On the contrary, negative correlations have also been reported [16]. According to a previous study investigating anthropometric indices, WHR remains a superior index to predict T2D [17]. As noted above, WHR and BMI are both positively correlated with HbA1c, a screening tool to detect Type 2 diabetes. However, to our knowledge, numerous research works in Taiwan have focused on BMI.

Exercise and HbA1c are essential for glycemic control in people with diabetes. In prior studies, resistance training was shown to significantly lower HbA1c levels in people with diabetes mellitus [18–20]. The results of meta-analyses indicated that both resistance and aerobic exercise significantly improved HbA1c levels among diabetic individuals [21]. Another systematic review and meta-analysis suggested that high- but not low-intensity resistance exercise was more beneficial for improving HbA1c levels in patients with diabetes mellitus (i.e., 0.61% vs. 0.23% reduction) [22]. HbA1c levels were substantially affected by resistance exercise compared to aerobic exercise [23]. Most of the studies describing correlations between changes in HbA1c and sociodemographic factors have focused on individuals with diabetes mellitus. In light of this, we evaluated the association of HbA1c with aerobic and resistance exercise in non-diabetic Taiwanese adults based on WHR.

## Materials and methods

### Participants, data source, and extraction

We obtained data from the TWB data source, which is the first large-scale biological database in Taiwan. Its purpose is to collect genetic, environmental, clinical, and lifestyle data and track the health of at least 200,000 adults for at least ten years. Additionally, this data source provides scholars and experts with important information about the causes and mechanisms of common diseases in Taiwan, which can lead to the improvement of health treatment policies and prevention strategies. Subjects in the biobank were between 30 and 70 years old and did not have a history of cancer. These participants had signed informed consents at various centers during assessment visits (between 2008 and 2019). Demographic and clinical data were available for 132,720 subjects in the biobank. We excluded those with diabetes mellitus (n = 12,711) and those with missing or incomplete information (n = 29,051). Our final analysis models included data from 90,958 subjects. We received ethical approval from the Institution Review Board of Chung Shan Medical University (CS1-21197).

### Outcome, exposure, and covariates

The primary outcome was HbA1c and WHR and exercise were the exposure variables. During recruitment, participants in TWB completed a questionnaire indicating how often, how long, and what kind of exercise they engaged in. Exercise was categorized as aerobic, resistance, and no exercise. Resistance exercise studied consisted of weight training, ball games, or a combination of both. On the other hand, aerobic physical activities included brisk walking, jogging, Taijiquan, rope jumping, gymnastics, yoga, Gigong, Chinese martial arts, swimming, hiking, biking, basketball, table tennis, soccer, badminton, tennis, golf, aerobic dance, ballroom dance, and hula hooping. Regular exercisers were those who participated in either aerobic or resistance exercise at least three times a week, lasting at least thirty minutes per session. The WHRs were categorized as normal or abnormal based on cut-off points: 0.92 for men and 0.88 for women as defined by the Health Promotion Administration, Ministry of Health and Welfare in Taiwan. It was measured as waist circumference (in cm) divided by hip circumference (in cm). HbA1c concentrations were measured using the automated XN-9000 hematology analyzer (Sysmex, Kakogawa, Japan).

### Statistical analysis

Differences in baseline characteristics stratified by normal WHR and abnormal WHR were tested using the χ2 test (for categorical variables, which were presented as absolute numbers [n] and percentages [%]) and student’s t-test (for continuous variables, which were explained as mean and standard error). We used multiple regression models to calculate β- coefficients of HBA1c with their 95% CIs. Baseline characteristics included exercise, HbA1c, WHR, sex, age, smoking, alcohol intake, hypertension, hyperlipidemia, and genetic variants. Data analyses were done by use of Plink version 1.9 and SAS 9.4 software (SAS Institute, Cary, NC, USA).

## Results

Table 1 shows the baseline characteristics of subjects with normal (n = 66,096, 72.67%) and abnormal (n = 24,862, 27.33%) WHR. The mean (±SE) age was 46.45 ± 0.041 years for those who had normal WHR and 51.63 ± 0.068 years for those with abnormal WHR. Effects of the study variables on HBA1c concentrations are shown in Table 2. In the multivariate model, lower HBA1c levels were associated with resistance exercise (β-coefficient = -0.027, 95% CI, -0.037 to -0.017; p<0.001) and aerobic exercise (β-coefficient = 0.018, 95% CI, -0.23 to -0.013; p<0.001). Higher HBA1c was associated with aging (β-coefficient = 0.009, 95% CI, 0.009 to 0.009; p<0.001), abnormal WHR (β-coefficient = 0.091, 95% CI, 0.086 to 0.096; p<0.001), current smoking (β-coefficient = 0.012, 95% CI, 0.005 to 0.020; p = 0.001), hypertension (β-coefficient = 0.046, 95% CI, 0.038 to 0.053; p = p<0.001), and hyperlipidemia (β-coefficient = 0.064, 95% CI, 0.055 to 0.074; p<0.001), and male sex (β-coefficient = 0.040, 95% CI, 0.036 to 0.045; p<0.001). We detected an interaction between exercise and WHR (p = 0.0181). In the multiple linear regression model stratified by WHR (Table 3), lower HbA1c was associated with aerobic exercise combined with either normal or abnormal WHR (β-coefficient = -0.019, 95% CI -0.024 to -0.013) and β-coefficient = -0.017, 95% CI -0.027 to -0.008, respectively (p<0.05). The β-coefficient was -0.028 (95% CI -0.039 to -0.017; p<0.001) for resistance exercise, coupled with normal WHR and -0.023 (95% CI, -0.046 to 0.000; p = 0.053) for resistance exercise, coupled with abnormal WHR. In the regression model with abnormal WHR and no exercise representing the reference group (Table 4), all combinations of either normal or abnormal WHR with exercise type (aerobic, resistance, or no exercise) were associated with decreased HBA1c levels. However, among the various subgroups, the lowest HBA1c levels were found in the ‘normal WHR and resistance exercise’ group (β-coefficient = -0.121, 95% CI, -0.132 to -0.109; p<0.001).

**Table 1:** Demographic characteristics of participants stratified by WHR.

Variables	Normal WHR	Abnormal WHR	p-value
	(n = 66,096)	(n = 24,862)	
**HbA1c, mean (±SE), %**	5.538(0.001)	5.684(0.002)	<0.001
**Exercise**			<0.001
No exercise	44913(67.95)	4025(55.66)	
Aerobic exercise	17917(27.11)	2884(39.88)	
Resistance exercise	3266(4.94)	322(4.45)	
**Sex**			<0.001
Women	47043(71.17)	16457 (66.19)	
Men	19053(28.83)	8405(33.81)	
**Age, mean (±SE), yr**	46.449(0.041)	51.630(0.068)	<0.001
**Smoking**			<0.001
Never	60179(91.05)	21516(86.54)	
Current	5917(8.95)	3346(13.46)	
**Alcohol drinking**			<0.001
Never	63244(9.69)	23131(93.04)	
Current	2852(4.31)	1731(6.96)	
**Hypertension**			<0.001
No	62322(94.29)	21006(84.49)	
Yes	3774(5.71)	3856(15.51)	
**Hyperlipidemia**			<0.001
No	63629(96.27)	22723(91.40)	
Yes	2467(3.73)	2139(8.60)	

Note: waist-to-hip ratio, HbA1c = glycosylated hemoglobin, yr = year, Ref = reference. Continuous data are presented as mean and standard error while categorical data are presented as absolute numbers (n) and percentages (%).

**Table 2:** Multiple linear regression showing effects of the study variables on HBA1c concentrations.

Variables	β value	p-value	95%C.I.
**Exercise (Ref: no exercise)**			
Aerobic exercise	-0.018	<0.001	-0.023, -0.013
Resistance exercise	-0.027	<0.001	-0.037, -0.017
**WHR (Ref: normal)**			
Abnormal	0.091	<0.001	0.086, 0.096
**Sex (Ref: Women)**			
Men	0.040	<0.001	0.036, 0.045
AGE	0.009	<0.001	0.009, 0.009
**Smoking (Ref: Never)**			
Current	0.012	0.001	0.005, 0.020
**Alcohol drinking (Ref: Never)**			
Current	-0.060	<0.001	-0.070, -0.050
**Hypertension (Ref: No)**			
Yes	0.046	<0.001	0.038, 0.053
**Hyperlipidemia (Ref: No)**			
Yes	0.064	<0.001	0.055, 0.074

Waist-to-hip ratio, HbA1c = glycosylated hemoglobin, Ref = reference, β = beta value, CI = 95% confidence interval.

**Table 3:** Multiple linear regression showing association of HbA1c with study variables based on WHR.

Variables	Normal WHR			Abnormal WHR		
	β value	p-value	95%C.I.	β value	p-value	95%C.I.
**Exercise (Ref: no exercise)**						
Aerobic exercise	-0.019	<0.001	-0.024, -0.013	-0.017	0.000	-0.027, -0.008
Resistance exercise	-0.028	<0.001	-0.039, -0.017	-0.023	0.053	-0.046, 0.000
**Sex (Ref: Women)**						
Men	0.050	<0.001	0.043, 0.054	0.017	0.001	0.007, 0.026
Age	0.010	<0.001	0.010, 0.010	0.008	<0.001	0.007, 0.008
**Smoking (Ref: Never)**						
Current	0.006	0.181	-0.003, 0.015	0.027	0.000	0.013, 0.041
**Alcohol consumption (Ref: Never)**						
Current	-0.058	<0.001	-0.070, -0.046	-0.063	<0.001	-0.080, -0.045
**Hypertension (Ref: No)**						
Yes	0.050	<0.001	0.039, 0.060	0.047	<0.001	0.035, 0.059
**Hyperlipidemia (Ref: No)**						
Yes	0.072	<0.001	0.059, 0.084	0.057	<0.001	0.042, 0.072

Note: WHR = waist hip ratio, HBA1c = glycosylated hemoglobin, Ref = reference, β = beta value, CI = 95% confidence interval.

**Table 4:** Association between HbA1c and different combinations of WHR and exercise using multivariate analyses.

Variables	β value	p-value	95%C.I.
**WHR & exercise habit** (Ref: abnormal WHR & no exercise)			
abnormal WHR & aerobic exercise	-0.028	<0.001	-0.037, -0.020
abnormal WHR & resistance exercise	-0.031	0.006	-0.052, -0.009
normal WHR & no exercise	-0.095	<0.001	-0.101, -0.090
normal WHR & aerobic exercise	-0.109	<0.001	-0.116, -0.103
normal WHR & resistance exercise	-0.121	<0.001	-0.132, -0.109
**Sex (Ref: Women)**			
Men	0.040	<0.001	0.036, 0.045
**Age**	0.009	<0.001	0.009, 0.009
**Cigarette smoking (Ref: Never)**			
Current	0.012	0.001	0.004, 0.020
**Alcohol drinking (Ref: Never)**			
Current	-0.060	<0.001	-0.069, -0.051
**Hypertension (Ref: No)**			
Yes	0.046	<0.001	0.038, 0.053
**Hyperlipidemia (Ref: No)**			
Yes	0.064	<0.001	0.055, 0.074

Note: WHR = waist hip ratio, HBA1c = glycosylated hemoglobin, Ref = reference, β = beta value, CI = 95% confidence interval.

## Discussion

Our analysis of TWB data indicated that compared with no exercise, aerobic and resistance exercises are associated with decreased levels of HbA1c in adults with no history of diabetes in Taiwan. However, reductions in HbA1c levels were greater in participants who performed resistance exercises (β-coefficient = -0.027 vs. -0.018). The same trend had also been noted among individuals with diabetes [23, 24] even though according to Yang and his team, it is yet to be proven whether resistance exercise differs from aerobic exercise regarding their effect on cardiovascular risk markers [21].

Additionally, our findings also indicated that normal compared to abnormal WHR raised HbA1c levels. It is important to note that the association of HbA1c with anthropometric parameters was also examined in populations without diabetes [25]. The results indicated that the degree of association varied. We detected an interaction between exercise and WHR in relation to HbA1c (p = 0.0181). Our stratified analysis (with ’abnormal WHR and no exercise’ as the reference group) showed that HbA1c levels decreased regardless of the subgroup. However, the greatest reductions occurred in the ‘normal WHR and resistance exercise’ group. In this group, the reduction in HbA1c was 0.121%. These results suggest that both WHR and exercise may be essential for regulating glycated hemoglobin. As far as we know, this is the first study to examine resistance and aerobic exercise in tandem with WHR among Taiwanese adults without diabetes. These findings add to our understanding of the factors controlling HbA1c concentrations, although further studies would help clarify their mechanisms.

In the general model, we also found that HbA1c was positively associated with age, abnormal compared to normal WHR, current compared to nonsmoking, and male compared to the female sex. Similar results have been previously reported among those with diabetes and prediabetes [26, 27]. In one of these studies [27], current smokers had higher HbA1c levels than non-smokers, by 0.08% (0.9 mmol/mol) and this was linked to oxidative stress. However, Nakagami and colleagues published contrary results for Japanese populations [28]. The WHR has been used to a lesser extent compared to other anthropometric parameters [15]. In line with previous findings [29, 30], we also found higher HbA1c values in participants with hypertension and hyperlipidemia.

We acknowledge a few limitations. First, exercise data were collected from self-reported questionnaires within the biobank; hence recall bias might have possibly occurred. Next, resistance exercise intensities have been investigated for glycemic biomarkers in patients with type 2 diabetes, where patients who performed high-intensity resistance exercise showed lower HbA1c levels than those who performed low-intensity resistance exercise [22]. However, in the current study, information was not available on the exercise intensity or volume.

## Conclusions

For the very first time, we have provided evidence that normal WHR together with resistance exercise may be associated with greater HbA1c reduction among non-diabetic individuals in Taiwan. Resistance exercise and WHR may be essential for strategies aimed at managing or improving HbA1c.
